# *Prunus lusitanica* L. Fruits: A Promising Underexploited Source of Nutrients with Potential Economic Value

**DOI:** 10.3390/foods12050973

**Published:** 2023-02-24

**Authors:** Ana Abraão, Manyou Yu, Irene Gouvinhas, Luís Ferreira, Amélia M. Silva, Raúl Domínguez-Perles, Ana Barros

**Affiliations:** 1 Centre for the Research and Technology of Agro-Environmental and Biological Sciences, (CITAB)/Institute for Innovation, Capacity Building and Sustainability of Agri-Food Production (Inov4Agro), University of Trás-os-Montes and Alto Douro (UTAD), Quinta de Prados, 5000-801 Vila Real, Portugal; 2 Department of Biology and Environment (DeBA-ECVA), University of Trás-os-Montes and Alto Douro (UTAD), Quinta de Prados, 5001-801 Vila Real, Portugal; 3 Phytochemistry and Healthy Food Lab (LabFAS), Centro de Edafología y Biología Aplicada del Segura (CEBAS), CSIC, University Campus of Espinardo, 25, 30100 Murcia, Spain

**Keywords:** *Prunus lusitanica* L., fresh fruits, wild variety, nutrients, agro-climatic conditions

## Abstract

In recent times, less-known fruit species have increasingly attracted worldwide attention and their health benefits are at the forefront. The fruits of plants from the genus *Prunus* are good sources of nutrients due to their economic, agronomic, and healthy values. However, *Prunus lusitanica* L., commonly known as Portuguese laurel cherry is considered an endangered species. Thus, the present work aimed to monitor the nutritional components of *P. lusitanica* fruits grown in three locations in northern Portugal for four consecutive years (2016–2019), using AOAC (Association of Official Analytical Chemists), spectrophotometric, and chromatographic analysis. The results evidenced the abundance of phytonutrients in *P. lusitanica*, such as proteins, fat, carbohydrates, soluble sugars, dietary fibre, amino acids, and minerals. It was also highlighted that the variation of nutritional components was relatively linked to the year factor, being especially relevant in the frame of the current changing climate, among others. These findings suggest that *P. lusitanica* L. deserves to be conserved and planted because of its food and nutraceutical applications. However, more detailed information on this rare plant species, such as phytophysiology, phytochemistry, bioactivity, pharmacology, etc., is certainly required for the design and development of appropriate uses and valorization alternatives for this species.

## 1. Introduction

**Figure 1:**
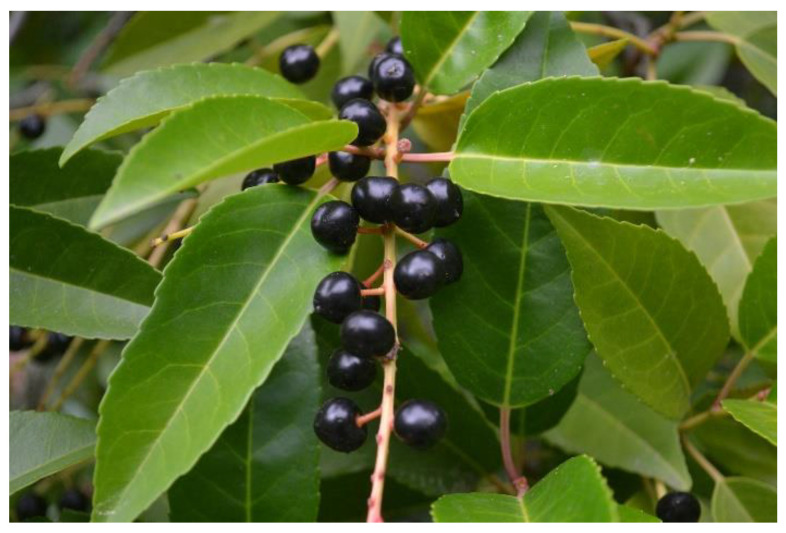
Representative image of *Prunus lusitanica* L. fresh fruits.

The genus *Prunus* L., which is classified under the family Rosaceae and subfamily Prunaceae or Amygdaloideae, has been one of the most economically and agronomically important plant genera in temperate regions [1]. This genus comprises deciduous or evergreen species, which are mostly used as edible fruits or ornamental plants [2]. Many edible fruits of *Prunus* species are commonly known as stone fruits (Figure 1), such as peach, plum, cherry, almond, etc., which consist of exocarp, mesocarp, and hardened endocarp with seed kernel [3]. *Prunus* species are considered healthy foods, due to their content of essential phytonutrients [2]. So, although their fruits provide a valuable content of simple sugar, closely related to the energy metabolism of cells, they also provide proteins, fat, organic acids, dietary fibre, and minerals, as well as bioactive phytochemicals in different concentrations, thus being potentially functional foods with multiple health benefits, namely improving digestion, enhancing the immune system, and alleviating metabolic disorders, such as obesity, hyperglycemia, hyperlipidemia, and hypertension [1,3,4,5,6,7,8]. For instance, orchard parameters starting from genotypes, climate change, soil condition, rootstocks, interstocks, canopy management, thinning, girdling, use of plant growth promoters, fertilizer and nutrient management, assisted pollination, age of tree and rootstock, etc., influence the nutritional, phytochemical, and sensorial quality of fruits and acceptance by consumers [1,9,10]. To the present date, despite this genus including approximately 400–430 species of shrubs and trees, only 98 *Prunus* species have been reported to be of any value [11].

*Prunus lusitanica* L., also known as Portuguese laurel cherry or Portugal laurel, is one of the lesser-known *Prunus* species, native to Portugal, Spain, Morocco, Macaronesia, and southwestern France with an Ibero–Maghrebian distribution in recent times [12]. It is regarded as a paleotropical relic species, which can adapt to different environments, thus helping to maintain the ecological balance and sustainable ecosystems [13]. Despite being listed under “least concern” on the “International Union for Conservation of Nature and Natural Resources (IUCN) Red List of Threatened Species” in 2017 [14], *P. lusitanica* specimens found on mainland Portugal were classified as “near threatened” on the “Red List of Vascular Flora of Mainland Portugal” in 2020 [15]. The communities of *P. lusitanica* in the central region of mainland Portugal are in sharp decline, with a loss of approximately 40% in the number of individuals and the quality of their habitat in the last 15 years, possibly due to the seasonal occurrence of rural fires and the expansion of invasive species [16]. Reducing the rate of biodiversity loss is one of humanity’s greatest challenges; moreover, increasing attention has been paid to the lesser-known fruit species because of their promising health benefits [17]. To our knowledge, to the present date, the fruits of *P. lusitanica* are not used/consumed in the human diet. Regarding its toxicity, although a certain degree of toxicity can empirically be attributed to the fruits of the species, they are considered edible fruits when they are completely ripe [18,19]. In this regard, Magalhães et al. [20] evaluated the cytotoxicity of *P. lusitanica* fruits in human fibroblasts and found that, at the concentrations studied, the extracts did not show toxicity. Until now, there has been a gap regarding the study of the nutritional composition of *P. lusitanica* fruits. To the best of our knowledge, the only study approaching the composition of these fruits has been our recently published article [21]. Therefore, it is quite urgent to explore its biochemical properties under different growth conditions, paving the way for its recovery and application/use in different industrial sectors.

Thus, the objectives of the present work were to characterize, for the first time, the phyto-nutritional content of *P. lusitanica* L. fruits grown at three different locations in northern Portugal, during four consecutive years (2016–2019) and to evaluate the relationship between the nutritional composition and the meteorological parameters measured during that period, aiming to address more attention to study this plant species, expand its cultivation and conservation, and highlight its potential utilization in the food, nutraceutical, and/or phytopharmaceutical industries.

## 2. Materials and Methods

### 2.1. Chemicals and Reagents

Sodium hydroxide, boric acid, sulfuric acid, ethanol, phosphate buffer, selenium, petroleum ether, glucose, α-amylase, amyloglucosidase, sodium acetate (anhydrous), triethylamine (TEA), and phosphoric acid were obtained from Sigma-Aldrich (Sigma-Aldrich Produktions GmbH, Steinheim, Germany). Authentic standards of the aminoacids used in the chromatographic analysis were purchased from Chem-Lab (Chem-Lab N.V., Zedelgem, Belgium), Sigma-Aldrich (Sigma-Aldrich, St. Louis, MO, USA), and Panreac (Panreac Química S.L.U., Barcelona, Spain), including L-alanine (Ala), L-arginine (Arg), L-asparagine (Asn), L-aspartic acid (Asp), glycine (Gly), L-glutamic acid (Glu), L-glutamine (Gln), L-histidine (His), L-isoleucine (Ileu), Leucine (Leu), L-phenylalanine (Phe), L-serine (Ser), L-threonine, (Thr), L-tryptophan, (Trp), L-tyrosine (Tyr), and L-valine (Val). The 6-Aminoquinolyl-*N*-hydroxysuccinimidyl carbamate (AQC) was purchased from Santa Cruz Biotechnology, Inc. (Heidelberg, Germany). Acetonitrile (UPLC grade), calcium disodium ethylene diamine tetraacetic acid (EDTA), sodium borate, and hydrochloric acid (HCl) were obtained from Panreac (Castelar del Vallés, Barcelona, Spain). Milli-Q purified water (Millipore, Bedford, MA, USA) was used for all the extraction and chromatographic analyses.

### 2.2. Collection and Preparation of Plant Material

Sampling took place in three different locations at the University of Trás-os-Montes and Alto Douro (UTAD) campus in northern Portugal, which is set in an eco-campus which includes one of Europe’s largest botanical gardens (Vila Real, Portugal). Plant material was collected for four years (2016–2019) when *P. lusitanica* L. fruits were fully matured according to the criteria of purple colour uniformity throughout the bunch (early October). *P. lusitanica* specimens studied in this study were duly identified in the botanical garden database at the UTAD.

Approximately 600 fruits were collected at random from trees naturally growing and located in three different areas of the UTAD eco-campus’s botanical garden. Sampling was always carried out in the same trees, which were marked at the beginning of the study, to guarantee that the same individuals would always be used over that period. Four trees were used in each location. *P. lusitanica* specimens grow naturally on campus, without anthropic action. No manipulation of the plant or soil was carried out. After collection, fruits were transported to the lab in refrigerated boxes and thoroughly mixed before being bulked into three well-mixed replicates. Whole fruits (pulp and stone) were frozen and freeze-dried at −80 °C (VirTis Benchtop Pro Freeze-drier with OmnitronicsTM, SP Industries, Inc., Warminster, PA, USA). Freeze-dried replicates were ground into a fine powder, stored hermetically, protected from light, and kept at −80 °C for further analysis (samples were stored for a maximum period of 2 weeks until analysis). Results are expressed in mg/100 g of fresh weight (fw).

The meteorological data corresponding to the seasons 2016–2019 are presented in Supplementary Table S1 and Figure S1. Meteorological data concerning the monthly accumulated precipitation (mm), maximum temperature (°C), minimum temperature (°C), and mean temperature (°C) in the seasons 2016, 2017, 2018, and 2019 were obtained from the E-OBS observational gridded dataset [22]. Daily air temperatures and precipitation were extracted from the gridbox corresponding to Vila Real.

### 2.3. Nutritional Composition of Prunus lusitanica Fruits

Lyophilized samples of *P. lusitanica* fruits were analysed in triplicate (*n* = 3), following the procedures described by the Association of Official Analytical Chemists (AOAC) [23], to determine the content of dry matter and ash (method 942.05), total protein (method 954.01), and total fat (method 920.39). Total dietary fibre analysis was carried out using a “Total Dietary Fiber Assay Kit”, based on AOAC method 985.29. Results are presented as the mean ± standard deviation (SD).

#### 2.3.1. Content of Dry Matter, Ash, and Moisture

To determine the dry matter content, 2.5 g of samples contained in the porcelain capsule were placed in an oven overnight at 105 °C, then cooled in the desiccator, and weighed for the dry residue. The dry matter (%) was determined as the ratio of dry residue weight to initial weight. The ash content was established by incinerating the dried samples at 550 °C in a muffle furnace (Umega Group AB, Ukmerge, Lithuania) for 3 h to a constant weight. The initial moisture content of fruits was determined by using a standard method according to the procedure described in the literature [24].

#### 2.3.2. Content in Total Protein, Total Fat, Carbohydrates, and Energy Value

For protein analysis, samples (0.2 g) after grinding and drying (air circulation at 65 °C) were digested in sulfuric acid (H_2_SO_4_) for 1 h at 420 °C in the presence of a selenium catalyst using a Buchi digestion unit. After acid digestion, with a Buchi distillation unit, the ammonia was distilled and collected in boric acid. Then, a titration was performed to measure the nitrogen content, as described in Kjeldahl’s method. Crude protein content was calculated as the nitrogen content × conversion factor of 6.25.

For lipid analysis, samples (3 g) were placed in an extraction thimble and extracted with 50 mL of petroleum ether using a Soxtec system HT6 (Tecator, Sweden). Extraction corresponded to 25 min of boiling in the organic solvent and thereafter 30 min of rinsing. Upon 10 min of solvent evaporation in the extraction system, the residue was dried at 105 °C overnight, cooled in a desiccator, and finally weighed. The crude content of total fat was calculated based on the dry weight of the samples.

The samples content in total carbohydrates was calculated by the formula:

“*carbohydrates* (g) = 100 (g) − *moisture* (g) − *ash* (g) − *protein* (g) − *fat* (g)”.

The energy vale of the fruits was calculated according to the methodology described by Crisan and Sands (1978) using the following formula: “*Energy value* (kcal/100 g) = (2.62 × *%protein*) + (8.37 × *%fat*) + (4.2 × *% carbohydrate*)” [25].

#### 2.3.3. Content of Dietary Fibre

A combination of enzymatic and gravimetric methods was used to determine the content of dietary fibre. Dried and defatted samples (1 g) of *P. lusitanica* fruits were mixed with 50 mL of phosphate buffer (pH 6.0) and subjected to gelatinization with 0.10 mL of heat-stable α-amylase (pH 6.0, 15 min, 95 °C). The mixture was cooled to room temperature (RT), adjusted at pH 7.5 (NaOH, 0.275 N), and digested with 0.10 mL of protease (pH 7.5, 30 min, 60 °C). Afterwards, the mixture was again cooled to RT, pH adjusted to 4.6 (HCl, 0.325 M), and digested with 0.10 mL of amyloglucosidase (pH 4.5, 30 min, 60 °C). After these digestion procedures, the beakers were covered with aluminium foil and incubated in water-bath (60 °C) with continuous agitation for 30 min. After adding 4 volumes of 95% ethanol to each beaker, the mixtures were maintained overnight to enable complete precipitation. The precipitates were filtered (using crucibles with a celite bed) and washed with 3 portions of 20 mL of 78% ethanol, 2 portions of 10 mL of 95% ethanol, and 2 portions of 10 mL of acetone. The crucibles containing the residues were dried in an air oven at 105 °C and then cooled in a desiccator. The weight was recorded. Three residues of each sample were used to determine the protein content by Kjeldahl nitrogen analysis, as specified by AOAC method 960.52 (1997) (6.25 was used as a conversion factor of ammonia to protein). The other three residues were incinerated at 525 °C for 5 h and cooled in the desiccator to RT. The dietary fibre content was obtained by subtracting the protein and ash content from the residue weight. 6 replicates of each sample and 6 blanks were performed (3 to the protein content and 3 to ashes determination).

#### 2.3.4. Content in Soluble Sugars

Soluble sugar samples were extracted with 80% ethanol (10 mL), using conic centrifuge tubes placed in the water-bath at 80 °C for 40 min. After cooling and centrifugation, the supernatant was transferred to small centrifuge tubes for the next analysis. Quantification of soluble sugars was based on the anthrone method using a standard curve established with a series of glucose solutions. Different amounts of extracts were transferred to new tubes, diluted accordingly, and were kept on ice during the whole reaction procedure. Then, 3 mL of anthrone reagent (prepared by dissolving 0.2 g of anthrone in 100 mL of 80% ethanol) was added to each tube, and the tubes were then placed in a water bath at 100 °C for 10 min. Finally, the samples were cooled to room temperature and the absorbance was read at 625 nm [26].

### 2.4. Amino Acids Analysis

For the extraction of free amino acids [27], the powdered samples (25 mg) were mixed with 5 mL of HCl (6 M) in a tube, then sealed with a cap and kept at 110 °C for 24 h. Then, samples were cooled to RT, pH was adjusted to 2.0 with NaOH, and transferred to volumetric flasks. Afterwards, 1 mL of internal standard solution (IS, L-norvaline, 5 mM) was added to the flask and the volume was adjusted to 50 mL with H_2_O. Each aliquot (1 mL) was subsequently filtered through a 0.22 μm filter and kept for further processing.

The pre-column derivatization was conducted with AQC as described by Batlazar et al. [28] with some modifications. The derivatization procedures consisted of adding 35 μL of borate buffer and 10 μL of AQC to 5 μL of standard or sample, in sequence. The capped mixture was kept at 50 °C for 10 min and was then placed in the auto-sampler at 10 °C.

Identification and quantification of amino acids of *P. lusitanica* fruits were performed by high-performance liquid chromatography with fluorescence detection (HPLC-FLD) system (Thermo Fisher Scientific Inc., Waltham, MA, USA), equipped with an RS quaternary pump, a WPS-3000RS auto sampler, a TCC-3000RS column holder, an FLD-3400RS fluorescence, and an ACE 5 C18 (5 μm, 150 mm × 4.6 mm i.d.) column (Advanced Chromatography Technologies Ltd., Aberdeen, UK) [29]. The mobile phases employed were: 140 mM sodium acetate, 17 mM TEA, 1 mM EDTA in water, pH 4.95 (phase A); acetonitrile/distilled water (60:40 *v*/*v*) (phase B); and water (phase C). The flow rate and injection volume were 1 mL/min and 5 μL, respectively. The employed linear gradient scheme (t, in min; %B; %C) was (0; 0%; 0%), (40; 33%; 7%), (48; 40%; 0%), and (53.5; 100%; 0%). Interpretation of the results was performed with Chromeleon Version 7.2 software (Thermo Fisher Scientific Inc., Waltham, MA). Amino acids were identified by comparison with authentic standards and were quantified according to calibration curves that were freshly prepared and analysed on every day of analysis.

### 2.5. Quantification of Mineral Composition

Eight mineral elements including Ca, Cu, Fe, K, Mg, Mn, Na, and Zn were determined. For each element determination, 0.5 g of lyophilized sample of *P. lusitanica* fruits was weighed in triplicate in digestion tubes. Digestion of the samples was performed with the method described by Gouvinhas et al. [30]. Concentrated HNO_3_ (2 mL) and H_2_O_2_ (1 mL, 30%) were added to the samples. Thereafter, the tubes were shaken and pre-digested overnight at RT. Subsequently, they were placed in a block heater with a glass sphere in each tube, thus avoiding evaporation. Initially, the samples were heated to 60 °C and then, temperature was gradually increased to 150 °C. Then, samples were digested to release all the organic matter, and when the mixture became clear and colourless, the glass spheres were removed, demonstrating that the HNO_3_ was completely evaporated. After cooling to RT, 10 mL of HNO_3_ matrix solution (1.5 mL of acid into 1 L of distilled water) was added and the samples were shaken.

Na and K quantification was performed by Flame Atomic Emission Spectrometry (FAES), while the quantification of Ca, Mg, Cu, Fe, Zn, and Mn was determined by flame atomic absorption spectrometry (FAAS) (Thermo scientific ICE 3000). Before the quantification of each element, calibration was carried out using mixed standards prepared in HNO_3_ (1.0 M), with five concentrations being selected, according to the expected for each element, plus the blank.

### 2.6. Statistical Analysis

All the assays were carried out in triplicate (*n* = 3) and results are expressed as mean ± standard deviation (SD). The statistical differences were obtained through analysis of variance (ANOVA) and a multiple range test (Tukey’s test) for a *p* < 0.05. Heat mapping of the Pearson´s correlations (commonly used to express the strength between two continuous variables, which is useful for demonstrating how the response variables are mathematically related and to understand the proportion of the fluctuation of one variable that was predictable from the other variable) with the respective statistical significances was performed in the software OriginPro 2022 v.9.9.0.225, to understand the nature and degree of inter-relationship among the parameters analysed and the meteorological data.

## 3. Results

### 3.1. Nutritional Composition of P. lusitanica Fruits Harvested from Different Locations in Each Year

Samples of *Prunus lusitanica* fruits collected in four years were separately evaluated to obtain the nutritional composition, including the energy value, content of ash, moisture, protein, total fat, available carbohydrates, dietary fibre, soluble sugars, amino acids (essential and non-essential), and minerals, as shown in Table 1, Table 2, Table 3 and Table 4.

Table 1 shows the nutritional composition of *P. lusitanica* fruits grown in different locations in 2016. Significant differences in the energy value of the fruits were observed between locations, with locations one and two presenting significantly higher values than location three. Concerning the basic nutrients, generally significant differences were observed between locations, with fruits from location one being characterized by the highest contents of almost all the analysed parameters, except in the cases of total fat and moisture. Regarding the amino acids (aa) content, nine essential aa (His, Arg, Thr, Val, Lys, Ile, Leu, Phe, Trp) and nine non-essential aa (Asp + Asn, Ser, Glu + Gln, Gly, Ala, Pro, Tyr) were identified and quantified. From the essential aa, Arg and Leu were the ones with the highest expression regardless of the location, with fruits from location two presenting the highest contents. In the case of the non-essential aa, Glu + Gln and Tyr were the ones that stood out. Considering the sum of the aa for each location, we can verify, through the analysis of Table 1. that fruits from location two presented significantly higher aa content than the ones from the other locations (trend verified in all amino acids except Tyr and Trp, whose contents were higher at location one, but not significantly different from those obtained at location two). Finally, regarding the total mineral content, following the trend observed in case the aa, fruits from location two presented the highest contents, followed by those from location one, with K, Na, and Fe being the ones with the highest contribution for all the locations, while Mn was the one present at the lowest content, with no significant differences between locations.

Regarding the results obtained for samples collected in 2017 (Table 2), the energy value of the fruits collected at location one stood out from the others, as they were significantly higher. Concerning basic nutrients (except for dietary fibre and soluble sugar), significant differences were observed between locations. For amino acids, a behaviour similar to that of the samples from 2016 was found for those present at the highest concentration (Arg and Leu in the case of essential and Glu + Gln and Tyr in the case of non-essential aa); however, in 2017, only Hys, Arg, Thr, Lys, Trp, and Gly presented significantly different contents between locations, with most of them found to be significantly higher in fruits from location two, except for Arg and Trp.

When analysing the mineral content, once again, those with the highest expression were K, Na, and Fe, while Mn was found at the lowest level. Additionally, significant differences between locations were observed for most minerals, except Cu and Mn. Furthermore, fruits from location one exhibited the highest content concerning individual minerals except for Na, whose content was significantly higher in location three.

Table 3 presents the nutritional values of *P. lusitanica* fruits grown in different locations in 2018. In terms of the energy value of the fruits harvested in the three different locations in 2018, it is possible to verify that significantly different values were observed in the three locations, with an emphasis on location one, followed by locations two and three. Concerning the basic nutrients, it is possible to observe that fruits from location one presented significantly higher ash, protein, and fat contents, while those from location three stood out regarding the remaining parameters analysed. Concerning the 18 identified amino acids, fruits from location one presented the highest amounts, except for Trp and Pro, which did not present significant differences between locations. The major amino acids were shown to be the same as in previous years, namely Arg, Leu, Glu + Gln, and Tyr. Concerning the minerals, similarly to what was observed for amino acids, the ones with the highest (K, Na, and Fe) and lowest (Mn) expression were the same as in the previous years of study, with significant differences found between locations for all the minerals under study, with fruits from location one presenting the highest contents for almost all of them.

Results regarding the nutritional composition of *P. lusitanica* fruits obtained from different locations in 2019 were presented in Table 4. In the case of the energy value of the fruits harvested in 2019, significant differences were observed between locations, with energy being highest in location two, followed by location one, and finally location three. Regarding the basic nutrients, significant differences were observed between locations, except for in ash content. Total fat, carbohydrates, and soluble sugar presented higher expression in fruits from location two, while moisture, protein, and dietary fibre were more prominent in fruits from location three. Concerning the amino acids, except for Thr, Lys, and Phe, significant differences were found between locations. Moreover, contrary to what was observed in the previous years of study, fruits from location three presented, in general, the highest amino acid contents (except for Ser and Gly, which were predominant in fruits from location two). In this regard, it is important to mention that Gly was not detected in *P. lusitanica* fruits harvested at location one, and contrary to what was observed in previous years, for essential amino acids, Lys proved to be the major aa, followed by Arg. Finally, regarding the minerals content, once again, fruits from location three stood out due to their highest content in the majority of the minerals analysed, except for Ca and Na, which in turn were higher in fruits from location two.

### 3.2. Comparative Analysis of Nutritional Composition of Fruits Grown under Different Locations or Years

To obtain a deeper insight into the location or year effects on the nutritional composition of *P. lusitanica* fruits, all the studied parameters were statistically compared between different locations throughout four years or between different years in the three locations, as shown in Table 5. The values presented in Table 5, concerning each location, were obtained by calculating the average of values recorded in each location in the 4 years of study (2016–2019), and the values for each year (Y2016, Y2017, Y2018, Y2019) were obtained by calculating the average values obtained for the three locations in each year. Overall, the effect of the location factor was not as noticeable as the effect of the year factor, except for in fat and Mn contents. Regarding the year factor, the opposite trend was verified, since significant differences were observed for most of the parameters analysed, except for the amino acids Asp + Asn, as well as for the trace element Mn.

From the analysis of Table 5, it can be seen that there was heterogeneity in results when a comparison was made between different years. The year 2016 never stood out for the highest contents in any of the analysed parameters. The year 2017 can be highlighted regarding the energy value of the fruits, and in terms of basic nutrients, for the higher content in ash, carbohydrates, and soluble sugars, as well as for the tendentially higher content in all non-essential amino acids (Asp + Asn, Ser, Glu + Gln, Gly, Ala, Pro, and Tyr) and in three of the nine minerals, namely Zn and the major ones K and Na, which contributed to significantly higher total mineral content in fruits from this year of study. In 2018, the highest contents of fat and dietary fibre were verified, as well as the essential aa Tyr and the minerals Cu and Fe. The year 2019 stood out for higher levels of moisture and protein, as well as for five of the nine essential amino acids (His, Arg, Thr, Val, and Lys) and also for the significantly higher total amino acid contents, providing levels almost 10 times higher relative to the additional years monitored. In fruits collected in 2019, the Ca and Mg levels also stood out.

### 3.3. Pearson’s Correlation Analysis between Nutritional Composition and Meteorological Data

**Figure 2:**
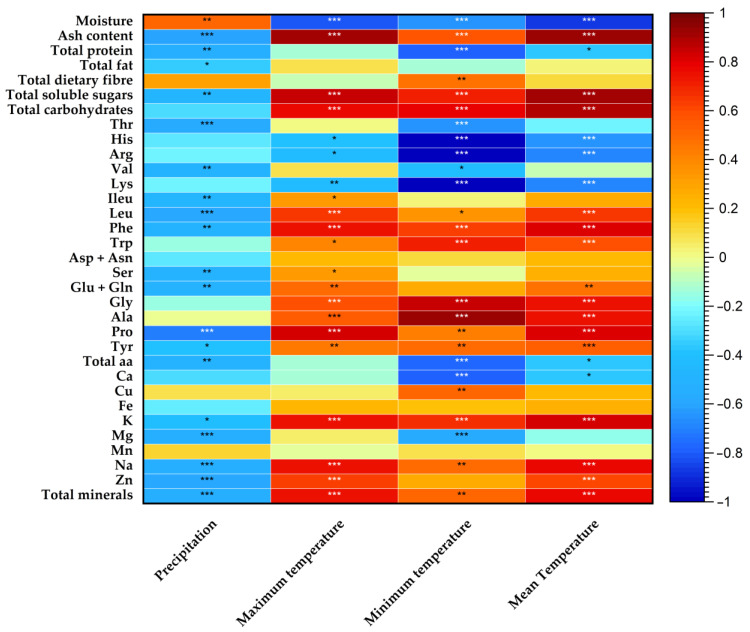
Heatmap of correlations between nutritional components of *P. lusitanica* fruits and meteorological data recorded monthly (accumulated precipitation, maximum, minimum and mean temperatures) from 2016 to 2019 as shown in Supplementary Table S1. Statistically significant correlations: * *p* < 0.05, ** *p* < 0.01, *** *p* < 0.001.

Since the year factor proved to be more influential in the analysed contents, compared to the location factor, a Pearson’s correlation coefficient was used to assess the correlation of the nutritional composition of *P. lusitanica* fruits with meteorological data from 2016 to 2019, as shown in Figure 2.

The results (Figure 2) showed that the precipitation negatively influenced the contents of the vast majority of the parameters analysed during the four years of study, since significant negative correlations were observed, with the exception of moisture, with which, as expected, it correlated in a significantly positive way. Regarding the temperature effect, significant positive and negative correlations were observed with the analysed parameters. Concerning the basic nutrients, a negative effect on the protein content was verified, especially for the minimum and mean temperature. Moreover, the remaining ash content, soluble sugar, and carbohydrates contents showed to be highly positively correlated with the three temperature parameters assessed. In the case of the amino acids content, interestingly, significant negative correlations were verified with the majority of the essential aa (His, Arg, Thr, Val, and Lys) and the total amino acids content and the temperature parameters, mainly with the minimum temperature. Conversely, significant positive correlations were observed for most aa, including all non-essential ones. Finally, regarding mineral elements, the minimum temperature exerted a significant negative effect on the Ca and Mg contents, whereas the mean temperature was only significantly negatively correlated with Ca. Regarding the mineral with the highest expression in our study (K), the three temperature parameters measured positively and significantly influenced its contents. Similar behaviour was verified for the total mineral content. In the cases of Cu, Na, and Zn, overall positive correlations were found with the temperature variables. Fe and Mn did not show any significant correlation with the studied meteorological parameters.

## 4. Discussion

This study aimed to investigate the variation of nutritional components, including the ash content, moisture, protein, fat, carbohydrates, dietary fibre, soluble sugar, amino acids, and minerals of *Prunus lusitanica* L. fruits grown under different locations during four years (from 2016 to 2019) in northern Portugal.

Because of their global distribution, plant species of the genus *Prunus* differ depending on climatic and soil conditions. Such variations in environmental conditions may also influence the chemical composition of these species, highlighting the presence of compounds that are considered nutraceuticals because they provide nutritional and health benefits [31]. Although *P. lusitanica* has a restricted habitat, edaphoclimatic conditions also influence the nutritional value of its fruits.

A nutrient is any substance obtained through food that the body can use for growth, maintenance, and repair, or it is simply a nourishing substance. They are divided into two categories: macronutrients, which provide energy, and micronutrients, which are essential for human health [32]. Observing Table 5, it is possible to verify that the significantly different energy values observed in all the years of the study appeared to be directly related mainly to the variation in the content of carbohydrates and soluble sugars, since in the years in which these components were present in higher concentrations, the energy value was higher, which would be expected, since the availability of energy from fruit and fruit-based products, including fruits belonging to the genus *Prunus*, is directly linked to the carbohydrate content, especially free sugars [33]. When compared with other fruits belonging to the same genus, the fruits of *P. lusitanica* presented an intermediate energy value, being higher than that presented by *P. avium*, *P. persica*, *P. armenica* [2,34,35], and lower than that shown by *P. dulcis* [36].

Total ash is the remained mass of dried fruits after being incinerated at the average temperature of 550 °C in the specific condition based on mass percentage and according to AOAC method 942.05. In the present study, although statistical significant differences were observed among locations in 2016, the small variation was negatively correlated with moisture content, but positively correlated with protein and carbohydrates content (Table 1). This can be further illustrated in Table 5. Compared with other *Prunus* species, lower ash contents were obtained in *Prunus divaricata* [37] and *Prunus avium* [34]; on the other hand, higher values were recorded in *Prunus korshinskyi* [38].

Moisture content has a significant impact on fruit characteristics such as physical appearance (shape, colour, etc.), texture, taste, and weight, in addition to other factors influencing shelf-life, freshness, quality, and resistance to bacterial and fungal contamination [39]. Our results indicated that water content represents the primary fraction of *P. lusitanica* fruits constituents, which is in agreement with other *Prunus* species fruits [10,37,40,41]. The differences in moisture between locations each year can be attributed to the rainfall, the age of trees that have different water demands, or sunlight, which may be affected by shelters [1,10]. Furthermore, the moisture content of fruits is affected by the nature of the fruit, as well as environmental factors such as water supply/precipitation [40]. Changes in the gradients between the water potentials of the roots, stems, leaves, and fruits, as well as environmental conditions such as the vapour pressure deficit, influence the water flow from the soil to the fruits. In fact, Measham et al. [42] reported a relation between the rainfall incidence and the water flow (sap) direction to the fruits through the tree. For instance, Peschel et al. [43] observed in *P. avium* that when the fruit surface was wet, greater water absorption was thought to occur, particularly during and after rainfall. We also verified this phenomenon in *P. lusitanica* fruits, since the content moisture correlated directly with the precipitation over the 4 years (Table S1 and Table 5), mainly in the month of October (month of fruit harvest that corresponds to the full maturity stage). 

Fruits are also a source of proteins, which are composed of amino acids. The high protein content in this study, ranging from 2.73 to 6.45 g/100 g fw, demonstrated that *P. lusitanica* fruits can also a source of proteins to be taken into account, being, to some extent, better than several fruits belonging to the same genus, namely *Prunus avium*, *Prunus dulcis*, *Prunus cerasus*, *Prunus laurocerasus*, *Prunus nepalensis*, *Prunus spinosa*, *Prunus armenica,* and *Prunus serotina* [44,45,46,47,48,49,50].

The healthy fat provided by fruits is an important part of a healthy diet, which can supply energy for the body and help to manage cholesterol levels [51]. Fat is an essential component of cell membranes and the natural wax present over the fruit surfaces or cuticle, and functions as carbon stores [52]. The significant differences in total fat content between locations and years (Table 5) suggested that both studied factors had effects on the fat content of *P. lusitanica* fruits, probably related to climate conditions. After comparing the *P. lusitanica* fruits fat content with others belonging to the same genus, we discovered that the amount present in our fruits was considerably lower than the ones found in several *P. dulcis* varieties [53,54], but higher than the observed, for instance, in *P. nepalensis* [50]. Carbohydrates, as an energy reservoir (yielding 4 kcal/g), are composed of polysaccharides, responsible for the structural formation of tissues and energy storage, and of oligosaccharides, and simple sugars [55]. As shown in this study, the carbohydrates content is the second most abundant fraction in *P. lusitanica* fruits, after water content. These results were consistent with other studies on fruits belonging to *Prunus* genus [3,44,56]. The significant variations of carbohydrates content between years rather than locations could imply that some climatic parameters such as sunlight, UV radiation, and temperature were probably relevant factors that impacted the photosynthesis of *P. lusitanica* fruits [57]. Additionally, from Table 5 it is evident that carbohydrates content was negatively correlated with moisture, which was an expected result. Carbohydrates content, in fruits harvested in Y2017, was on average the highest which coincided with the year of lowest rainfall during fruit ripening (August to October), while fruits from Y2019 and Y2016 had the lowest carbohydrates content having high rainfall in the same period in these years (Table S1). 

Soluble sugars are the main component of carbohydrates in fruits, and they can be found in the forms of fructose, glucose, sucrose, arabinose, and sorbitol [58], which are vital for plant structure and metabolism at the cellular and whole-organism levels. The content of soluble sugars evaluated in the present work accounted for over 50% of carbohydrates, which was relatively more linked to the year factor, namely to the lower precipitation and higher temperatures observed during the ripening stages (Table S1). Similar sugar levels were obtained by Saidani et al. [59] in *Prunus persica*; however, Guo et al. [60] obtained slightly lower values in peaches (*Prunus persica*) and nectarines (*Prunus persica* var. *nucipersica*), which were both immature and mature.

Dietary fibres, mainly classified into insoluble and soluble categories, are a group of carbohydrates, heterogeneous substances composed of cellulose, hemicellulose, pectins, lignins, gums, and polysaccharides [35,61], and represent carbohydrates that are not digested or absorbed in the small intestine. They exert direct physiological effects throughout the gastrointestinal tract and present a desirable influence on weight regulation, carbohydrates and lipid metabolism, and colon function, playing this way an important role in the prevention of diseases such as cardiovascular disease, colorectal cancer, lung cancer, obesity, and type 2 diabetes [50]. As an overall conclusion from the results of this study, it was found that this fruit can be considered an excellent source of dietary fibre because, according to Regulation (EC) n. 1924/2006, food systems containing at least 3 g of fibre per 100 g or at least 1.5 g of fibre per 100 kcal can be considered a source of fibre [62]. The dietary fibre levels obtained in our study were in the range of the ones observed in *Prunus spinosa* and *Prunus domestica* fruits [47,63,64], but higher than the ones reported in *Prunus nepalensis, Prunus dulcis,* and *Prunus avium* [34,50,65].

Amino acids may occur in free form or in non-protein compounds, which can contribute to both nutritional value and the taste of fruits [4,66]. They enhance the taste of other compounds and have specific tastes, which range from bitter (Leu, Phe, Trp, or Tyr) to sweet (Pro, Ala) and tasteless (Arg, Asp, Ileu, Lys, Ser, Thr, Val) [66,67]. The total levels of identified amino acids varied from 2802.00 mg/100 g fw (loc 3, 2016) to 6948.93 mg/100 g fw (loc 3, 2019); these were much higher than those obtained in other fruits such as *Prunus persica* [35,60,67,68], namely concerning the essential amino acids Val, Lys, Ileu, Leu, and Arg [35]. Lower amino acids content was also found in *Prunus avium* fruits [58]. In the current study, the significance of individual amino acids between locations was largely different among years; for instance, the concentration of Trp displayed statistical changes in the years 2016, 2017, and 2019, but presented no differences between 2017 and 2018. In addition, the insignificant variations in the content of Asp + Asn between both locations and years may suggest that the synthesis of these aa were not influenced by external factors [69]. Malnutrition is a widespread problem in many countries worldwide, where population growth has led to increasing demand for high-quality protein [70]. As a result, finding new and inexpensive sources of amino acids is critical; furthermore, the importance of wild and unconventional plant sources in human nutrition is recognized [71,72].

Minerals in foods play critical roles in the human body and are required to meet its physiological needs [73]. Some macro- and micro-elements can be found in the structure of teeth (Ca, P, and F) and bones (Ca, Mg, Mn, P, B, and F), whereas most micro-elements (Cu, Fe, Mn, Mg, Se, and Zn) play an important structural role in many enzymes [74]. In the present study, the mineral content was evaluated. K was the element presenting the highest levels in our study, followed by Na, regardless of the year and location. Other authors verified this trend in fruits belonging to other *Prunus* species [2,34,37,39,41,59,73,75,76,77]. In general, the levels obtained in our study regarding Ca and Mg were lower than those reported in the literature in fruits of other *Prunus* species, such as *Prunus padus* and *Prunus spinosa* [78]. In the case of Na, Fe, and Zn, the levels obtained in this study were higher than in other *Prunus*. For Mn, the contents were within the same range of values, as in other *Prunus*. It is worth noting that the Mn concentration of *P. lusitanica* fruits was not statistically variable between locations in the year 2016 (Table 1), 2017 (Table 2), and 2019 (Table 4), neither was it variable in different years (Table 5). Yet, the mean value of this component through the 4 investigated years showed significant differences among locations, which might be involved in mineral metabolism according to the soil conditions, since minerals in plants reflect the soil composition in which the plant is growing [79,80]. For instance, Sun et al. [81] verified that soil which had available N, K, Ca, Fe, and B content contributed the most to the quality of *Prunus persica* fruits.

According to our general findings, as aforementioned, a correlation heatmap between the nutritional composition of *Prunus lusitanica* fruits and the meteorological parameters during 2016–2019 was performed. Colour-coded heatmaps make it simple to identify variables that correlate positively or negatively [82]. Increasing studies have noted the effects of climatic factors on fruits’ nutrients [83,84,85,86]. Environmental conditions are a major factor affecting the variability of the nutritional composition of fruits and vegetables [87,88]. The significant change in nutrient levels is an indicator of climate change impact is growing, as evidenced by the assessment of the quality of vegetables and legumes under different climatic scenarios, as conducted by Scheelbeek et al. [89]. However, understanding the role of climatic factors in shifting the nutritional composition of foods and the consequences for human health is extremely challenging [90]. Temperature rise, atmospheric CO_2_, drought, and O_3_ concentrations, as well as other indirectly related climate change factors, will all affect quality, but the outcome (positive or negative) depends on species and a specific quality trait. On the one hand, it may improve some quality traits related to primary metabolism (e.g., photosynthesis), such as flavour associated with carbohydrates (e.g., sweetness), and improve biochemical pathways related to plant defence mechanisms, leading to an improvement in some nutritional traits (e.g., antioxidants), but, on the other hand, it may harm product appearance (e.g., visual disorders, malformations) and nutritional value related to proteins, minerals, and amino acids [91]. Several authors have studied the effect of the different weather variables on the quality and nutritional profile of fruits belonging to the *Prunus* genus reinforcing the results obtained in this study. For example, environmental factors such as day/night temperature have been shown to influence *Prunus persica* fruit ripening and nutritional quality at harvest [92]; in the same species, Iglesias et al. [93] also corroborated the influence of climatic factors associated with the year of study on the nutritional and qualitative characteristics of the fruits. López-Ortiz et al. [94] verified in their 2-year study (2004 and 2005) in different *Prunus dulcis* cultivars that fat content was influenced by the year factor, and similar findings were obtained by Čolić et al. [95]. In turn, Lakatos et al. [96], in their studies on *Prunus cerasus* fruits, in good agreement with our results, found that precipitation was negatively correlated with the dry matter content of the fruits, whereas the sugar concentration was positively correlated with the difference between daytime and night-time temperatures, as well as night temperature.

## 5. Conclusions

The present study described the evaluation and variation of nutritional constituents of *P. lusitanica* fruits grown at three locations in northern Portugal during four consecutive years (2016–2019). To the best of our knowledge, this work describes, for the first time, that *P. lusitanica* fruits are a rich source of phytonutrients, such as proteins, fat, carbohydrates, soluble sugars, dietary fibre, amino acids, and minerals, in many cases surpassing conventional fruits widely consumed, demonstrating that this species is worth being preserved and cultivated. Our results also highlighted that the variation of its nutritional components was linked to the year factor and correlated to the environmental variables (precipitation and temperature), which might be correlated to climate change, among other factors. More research into changes in the quality and yield of fruit and vegetable crops as a result of global climate change should be prioritized, as they will become increasingly linked to food scarcity. The nutrient profile of *P. lusitanica* fruits observed during this study emphasizes the possible inclusion of these fruits in different industrial sectors, popularizing and recommending them for commercial exploitation. However, more detailed information on this plant species such as phytophysiology, phytochemistry, bioactivity, toxicology, pharmacology, etc., is required to identify the near-future applications of *P. lusitanica* products to create healthy, nutritious, and potentially functional food products for humans.

## Figures and Tables

**Table 1:** Nutritional composition of *P. lusitanica* fruits grown under different locations in 2016.

Nutritional Composition	Location			*p*-Value
	Location 1	Location 2	Location 3	
**Energy value** **(kcal/100 g)**	159.37 ± 0.35 ^b^	160.2 ± 0.09 ^b^	133.9 ± 0.77 ^a^	<0.001
**Basic nutrients** **(g/100 g fw)**				
Ash	1.62 ± 0.00 ^c^	1.51 ± 0.00 ^b^	1.36 ± 0.00 ^a^	<0.001
Moisture	62.51 ± 0.00 ^a^	63.28 ± 0.00 ^b^	69.96 ± 0.00 ^c^	<0.001
Protein	3.26 ±0.12 ^b^	3.01 ±0.13 ^b^	2.73 ± 0.01 ^a^	0.002
Fat	2.33 ± 0.01 ^a^	3.11 ± 0.12 ^b^	3.32 ± 0.19 ^b^	<0.001
Carbohydrates	30.29 ±0.13 ^c^	29.10 ± 0.24 ^b^	22.63 ± 0.20 ^a^	<0.001
Dietary Fibre	9.80 ± 0.31 ^b^	10.26 ± 0.06 ^b^	8.22 ± 0.14 ^a^	<0.001
Soluble Sugar	17.89 ± 0.15 ^b^	17.67 ± 0.10 ^b^	14.40 ± 0.02 ^a^	<0.001
**Amino acids** **(mg/100 g fw)**				
**Essential amino acids**				
His	34.98 ± 2.32 ^b^	36.28 ± 1.88 ^b^	30.24 ± 0.78 ^a^	0.014
Arg	134.28 ± 7.55 ^a^	172.56 ± 20.61 ^b^	130.86 ± 4.88 ^a^	0.014
Thr	47.82 ± 1.11 ^bc^	53.23 ± 4.46 ^c^	43.14 ± 3.78 ^a^	0.032
Val	74.40 ± 2.93 ^bc^	87.09 ± 10.38 ^c^	70.34 ± 3.57 ^a^	0.047
Lys	78.70 ± 7.22 ^b^	128.25 ± 7.62 ^c^	60.77 ± 5.97 ^a^	<0.001
Ileu	59.85 ± 0.65 ^bc^	67.24 ± 6.22 ^c^	55.53 ± 3.76 ^a^	0.038
Leu	146.71 ± 4.31 ^bc^	159.51 ± 10.17 ^c^	134.78 ± 4.28 ^a^	0.013
Phe	101.28 ± 6.92 ^bc^	108.21 ± 2.33 ^c^	90.32 ± 3.00 ^a^	0.008
Trp	13.92 ± 1.03 ^a^	13.67 ± 0.12 ^a^	12.62 ± 0.52 ^a^	N.s.
**Non-essential amino acids**				
Asp + Asn	580.63 ± 37.10 ^b^	700.49 ± 54.32 ^c^	467.84 ± 25.95 ^a^	0.001
Ser	55.32 ± 2.12 ^a^	71.65 ± 8.21 ^b^	52.04 ± 3.86 ^a^	0.009
Glu + Gln	720.28 ± 34.24 ^a^	828.28 ± 89.28 ^a^	691.79 ± 16.81 ^a^	N.s.
Gly	66.46 ± 8.97 ^a^	81.43 ± 2.66 ^c^	71.22 ± 2.74 ^bc^	0.043
Ala	83.94 ± 2.25 ^a^	95.80 ± 11.78 ^a^	83.06 ± 8.67 ^a^	N.s.
Pro	118.38 ± 4.74 ^b^	122.65 ± 7.74 ^b^	77.45 ± 3.47 ^a^	<0.001
Tyr	828.70 ± 24.38 ^b^	814.62 ± 0.47 ^b^	730.01 ± 17.03 ^a^	<0.001
**Total aa**	3145.66 ± 60.86 ^b^	3540.96 ± 129.65 ^c^	2802.00 ± 66.82 ^a^	<0.001
**Minerals** **(mg/100 g fw)**				
Ca	0.72 ± 0.01 ^a^	0.78 ± 0.01 ^b^	0.88 ± 0.03 ^c^	<0.001
Cu	0.40 ± 0.01 ^b^	0.41 ± 0.01 ^b^	0.27 ± 0.02 ^a^	<0.001
Fe	1.29 ± 0.15 ^a^	1.36 ± 0.03 ^a^	1.36 ± 0.09 ^a^	N.s.
K	4.89 ± 0.04 ^b^	4.81 ± 0.04 ^b^	3.49 ± 0.10 ^a^	<0.001
Mg	0.88 ± 0.03 ^b^	0.93 ± 0.01 ^b^	0.79 ± 0.03 ^a^	0.001
Mn	0.16 ± 0.00 ^a^	0.18 ± 0.00 ^a^	0.19 ± 0.02 ^a^	0.057
Na	2.36 ± 0.15 ^b^	2.36 ± 0.05 ^b^	2.03 ± 0.03 ^a^	0.006
Zn	0.51 ± 0.00 ^b^	0.48 ± 0.05 ^b^	0.40 ± 0.02 ^a^	0.013
**Total minerals**	11.22 ± 0.39 ^b^	11.31 ± 0.05 ^b^	9.40 ± 0.16 ^a^	<0.001

Values are presented as the mean ± SD (*n* = 3) for the content of each nutritional composition at different locations. Mean values followed by different lowercase letters report significant differences between distinct locations, according to the analysis of variance (ANOVA) and Tukey’s multiple range test. N.s., not significant.

**Table 2:** Nutritional composition of *P. lusitanica* fruits grown under different locations in 2017.

Nutritional Composition	Location			*p*-Value
	Location 1	Location 2	Location 3	
**Energy value** **(kcal/100 g)**	194.73 ± 1.8 ^b^	190.72 ± 0.17 ^a^	189.43 ± 1.94 ^a^	0.013
**Basic nutrients** **(g/100 g fw)**				
Ash	2.00 ± 0.00 ^a^	2.00 ± 0.00 ^a^	2.09 ± 0.00 ^b^	0.021
Moisture	55.87 ± 0.00 ^b^	55.73 ± 0.00 ^a^	56.22 ± 0.00 ^c^	<0.001
Protein	4.82 ± 0.02 ^c^	3.79 ± 0.04 ^a^	4.22 ± 0.15 ^b^	<0.001
Fat	5.32 ± 0.41 ^b^	3.43 ± 0.07 ^a^	3.94 ± 0.35 ^a^	<0.001
Carbohydrates	31.99 ± 0.39 ^a^	35.05 ± 0.12 ^c^	33.53 ± 0.19 ^b^	<0.001
Dietary Fibre	8.76 ± 0.64 ^a^	8.81 ± 0.61 ^a^	9.34 ± 0.15 ^a^	N.s.
Soluble Sugar	21.24 ± 0.12 ^a^	21.24 ± 0.10 ^a^	21.56 ± 0.18 ^a^	N.s.
**Amino acids** **(mg/100 g fw)**				
**Essential aminoacids**				
His	47.22 ± 5.25 ^a^	72.46 ± 1.68 ^b^	45.31 ± 5.80 ^a^	<0.001
Arg	208.91 ± 10.83 ^ab^	173.02 ±9.04 ^a^	220.54 ± 25.55 ^b^	0.032
Thr	71.49 ± 0.53 ^a^	98.41 ± 7.05 ^b^	74.38 ± 8.34 ^a^	0.004
Val	117.20 ± 5.58 ^a^	126.67 ± 13.04 ^a^	117.11 ± 5.49 ^a^	N.s.
Lys	104.66 ± 5.05 ^a^	141.78 ± 19.91 ^b^	94.06 ± 12.88 ^a^	0.013
Ileu	97.15 ± 14.02 ^a^	80.48 ± 7.10 ^a^	96.96 ± 10.26 ^a^	N.s.
Leu	215.17 ± 14.64 ^a^	204.90 ± 22.41 ^a^	216.13 ± 8.05 ^a^	N.s.
Phe	142.85 ± 9.56 ^a^	132.31 ± 6.02 ^a^	149.15 ± 6.93 ^a^	N.s.
Trp	20.33 ± 1.65 ^b^	18.37 ± 0.49 ^ab^	16.90 ± 0.84 ^a^	0.025
**Non-essential aminoacids**				
Asp + Asn	830.27 ± 50.80 ^a^	731.35 ± 108.70 ^a^	701.44 ± 77.79 ^a^	N.s.
Ser	103.32 ± 10.71 ^a^	88.74 ± 11.52 ^a^	82.22 ± 4.74 ^a^	N.s.
Glu + Gln	1170.26 ± 59.03 ^a^	968.91 ± 42.41 ^a^	996.72 ± 135.70 ^a^	N.s.
Gly	124.85 ± 13.64 ^a^	170.93 ± 9.93 ^b^	107.30 ± 3.14 ^a^	<0.001
Ala	136.04 ± 8.50 ^a^	119.08 ± 13.60 ^a^	116.13 ± 9.67 ^a^	N.s.
Pro	214.97 ± 14.03 ^a^	253.87 ± 19.68 ^a^	238.20 ± 22.36 ^a^	N.s.
Tyr	1124.83 ± 67.64 ^a^	1033.12 ± 32.84 ^a^	1002.93 ± 38.09 ^a^	N.s.
**Total aa**	472.53 ± 111.60 ^b^	4414.43 ± 225.90 ^ab^	4275.47 ± 185.82 ^a^	0.035
**Minerals** **(mg/100 g fw)**				
Ca	0.83 ± 0.01 ^b^	0.82 ± 0.00 ^b^	0.76 ± 0.04 ^a^	0.013
Cu	0.44 ± 0.00 ^a^	0.38 ± 0.04 ^a^	0.40 ± 0.00 ^a^	N.s.
Fe	1.83 ± 0.06 ^b^	1.82 ± 0.00 ^b^	1.67 ± 0.03 ^a^	0.006
K	6.03 ± 0.02 ^c^	5.85 ± 0.08 ^b^	5.71 ± 0.01 ^a^	<0.001
Mg	1.06 ± 0.02 ^b^	0.99 ± 0.01 ^a^	0.99 ± 0.01 ^a^	<0.001
Mn	0.17 ± 0.00 ^a^	0.17 ± 0.02 ^a^	0.15 ± 0.00 ^a^	N.s.
Na	2.79 ± 0.08 ^a^	2.98 ± 0.07 ^a^	3.94 ±0.08 ^b^	<0.001
Zn	0.89 ± 0.07 ^b^	0.61 ± 0.05 ^a^	0.73 ± 0.02 ^a^	0.002
**Total minerals**	14.04 ± 0.13 ^b^	13.62 ± 0.12 ^a^	14.35 ± 0.02 ^c^	<0.001

Values are presented as the mean ± SD (*n* = 3) for the content of each nutritional composition at different locations. Mean values followed by different lowercase letters report significant differences between distinct locations, according to the analysis of variance (ANOVA) and Tukey’s multiple range test. N.s., not significant.

**Table 3:** Nutritional composition of *P. lusitanica* fruits grown under different locations in 2018.

Nutritional Composition	Location			*p*-Value
	Location 1	Location 2	Location 3	
**Energy value** **(kcal/100 g)**	181.78 ± 0.22 ^c^	165.72 ± 0.68 ^b^	157.93 ± 0.18 ^a^	<0.001
**Basic nutrients (g/100 g fw)**				
Ash	1.75 ± 0.04 ^b^	1.48 ± 0.03 ^a^	1.50 ± 0.04 ^a^	<0.001
Moisture	60.08 ± 0.04 ^a^	62.06 ± 0.04 ^b^	63.00 ± 0.25 ^c^	<0.001
Protein	4.14 ± 0.03 ^c^	4.04 ± 0.02 ^b^	3.86 ± 0.01 ^a^	<0.001
Total Fat	5.93 ± 0.08 ^c^	4.00 ± 0.18 ^b^	2.94 ± 0.05 ^a^	<0.001
Carbohydrates	28.09 ± 0.11 ^a^	28.42 ± 0.19 ^b^	28.71 ± 0.06 ^b^	0.004
Dietary Fibre	10.81 ± 0.07 ^b^	8.60 ± 0.28 ^a^	12.08 ± 0.15 ^c^	<0.001
Soluble Sugar	17.89 ± 0.05 ^b^	17.51 ± 0.10 ^a^	18.25 ± 0.03 ^c^	<0.001
**Amino acids (mg/100 g fw)**				
**Essential**				
His	45.34 ± 0.67 ^b^	42.39 ± 0.38 ^ab^	37.07 ± 3.69 ^a^	0.010
Arg	223.72 ± 28.29 ^b^	207.40 ± 8.38 ^b^	145.15 ± 8.95 ^a^	0.004
Thr	93.28 ± 7.19 ^c^	66.29 ± 1.83 ^b^	52.58 ± 5.38 ^a^	<0.001
Val	121.46 ± 3.10 ^b^	119.00 ± 6.61 ^b^	87.36 ± 3.89 ^a^	<0.001
Lys	103.97 ± 4.03 ^c^	78.28 ± 3.66 ^b^	66.16 ± 0.06 ^a^	<0.001
Ileu	101.31 ± 8.89 ^c^	81.94 ± 5.98 ^b^	65.05 ± 0.64 ^a^	0.001
Leu	212.56 ± 2.05 ^c^	178.81 ± 1.75 ^b^	153.07 ± 0.67 ^a^	<0.001
Phe	135.65 ± 0.70 ^c^	123.52 ± 0.21 ^b^	104.93 ± 2.83 ^a^	<0.001
Trp	20.50 ± 1.21 ^a^	20.33 ± 1.36 ^a^	19.21 ± 1.10 ^a^	N.s.
**Non-essential**				
Asp + Asn	821.32 ± 23.68 ^b^	825.12 ± 36.33 ^b^	572.06 ± 37.09 ^a^	<0.001
Ser	110.29 ± 3.89 ^c^	72.23 ± 3.50 ^b^	56.75 ± 1.56 ^a^	<0.001
Glu + Gln	1108.10 ± 14.06 ^b^	930.47 ± 6.99 ^a^	870.82 ± 77.59 ^a^	0.002
Gly	137.84 ± 7.27 ^b^	146.23 ± 10.99 ^b^	82.22 ± 6.47 ^a^	<0.001
Ala	134.73 ± 2.94 ^b^	130.94 ± 1.19 ^b^	90.78 ± 4.69 ^a^	<0.001
Pro	165.59 ± 4.11 ^a^	160.01 ± 4.97 ^a^	164.35 ± 0.11 ^a^	N.s.
Tyr	1101.32 ± 30.20 ^b^	1099.26 ± 56.64 ^b^	983.24 ± 51.08 ^a^	0.036
**Total aa**	4637.00 ± 39.94 ^c^	4282.22 ± 73.00 ^b^	3550.80 ± 121.30 ^a^	<0.001
**Minerals (mg/100 g fw)**				
Ca	0.70 ± 0.04 ^b^	0.62 ± 0.01 ^a^	0.65 ± 0.00 ^ab^	0.013
Cu	0.58 ± 0.02 ^b^	0.38 ± 0.05 ^a^	0.57 ± 0.00 ^b^	<0.001
Fe	2.09 ± 0.01 ^c^	1.77 ± 0.06 ^b^	1.50 ± 0.05 ^a^	<0.001
K	5.62 ± 0.01 ^c^	4.83 ± 0.04 ^b^	4.36 ± 0.29 ^a^	<0.001
Mg	1.09 ± 0.02 ^b^	0.88 ± 0.03 ^a^	0.91 ± 0.03 ^a^	<0.001
Mn	0.21 ± 0.01 ^b^	0.10 ± 0.00 ^a^	0.20 ± 0.02 ^b^	<0.001
Na	2.68 ± 0.01 ^b^	2.86 ± 0.06 ^c^	2.28 ± 0.06 ^a^	<0.001
Zn	0.75 ± 0.03 ^c^	0.59 ± 0.00 ^b^	0.42 ± 0.02 ^a^	<0.001
**Total minerals**	13.71 ± 0.01 ^c^	12.03 ± 0.15 ^b^	10.89 ± 0.42 ^a^	<0.001

Values are presented as the mean ± SD (*n* = 3) for the content of each nutritional composition at different locations. Mean values followed by different lowercase letters report significant differences between distinct locations, according to the analysis of variance (ANOVA) and Tukey’s multiple range test. N.s., not significant.

**Table 4:** Nutritional composition of *P. lusitanica* fruits grown under different locations in 2019.

Nutritional Composition	Location			*p*-Value
	Location 1	Location 2	Location 3	
**Energy value** **(kcal/100 g)**	137.64 ± 0.68 ^b^	145.89 ± 1.11 ^c^	120.79 ± 0.63 ^a^	<0.001
**Basic nutrients** **(g/100 g fw)**				
Ash	1.34 ± 0.04 ^a^	1.37 ± 0.04 ^a^	1.32 ± 0.01 ^a^	N.s.
Moisture	68.86 ± 0.04 ^b^	66.50 ± 0.02 ^a^	69.71 ± 0.03 ^c^	<0.001
Protein	4.99 ± 0.02 ^a^	5.64 ± 0.12 ^b^	6.45 ± 0.13 ^b^	<0.001
Total Fat	4.84 ± 0.15 ^b^	4.95 ± 0.20 ^b^	2.78 ± 0.13 ^a^	<0.001
Carbohydrates	19.97 ± 0.15 ^a^	21.54 ± 0.17 ^b^	19.73 ± 0.18 ^a^	<0.001
Dietary Fibre	7.76 ± 0.16 ^b^	6.68 ± 0.18 ^a^	9.97 ± 0.10 ^c^	<0.001
Soluble Sugar	14.16 ± 0.10 ^b^	15.08 ± 0.03 ^c^	13.32 ± 0.11 ^a^	<0.001
**Amino acids** **(mg/100 g fw)**				
**Essential aminoacids**				
His	252.79 ± 16.27 ^a^	272.93 ± 25.55 ^ab^	321.65 ± 15.86 ^b^	0.013
Arg	1189.02 ± 20.02 ^a^	1292.62 ± 41.84 ^a^	1500.39 ± 74.59 ^b^	<0.001
Thr	87.22 ± 12.54 ^a^	108.32 ± 15.33 ^a^	114.33 ± 4.31 ^a^	N.s.
Val	70.83 ± 9.04 ^a^	158.03 ± 0.36 ^b^	170.86 ± 6.77 ^b^	<0.001
Lys	1256.57 ± 152.30 ^a^	1245.17 ± 126.50 ^a^	1323.38 ± 77.04 ^a^	N.s.
Ileu	58.38 ± 1.28 ^a^	62.72 ± 2.90 ^a^	112.35 ± 3.34 ^b^	<0.001
Leu	135.71 ± 14.57 ^a^	147.72 ± 16.19 ^ab^	183.15 ± 17.22 ^b^	0.026
Phe	86.27 ± 4.73 ^a^	86.92 ± 9.14 ^a^	96.58 ± 8.09 ^a^	N.s.
Trp	8.58 ± 0.22 ^a^	9.66 ± 1.51 ^a^	14.15 ± 0.28 ^b^	<0.001
**Non-essential aminoacids**				
Asp + Asn	460.08 ± 58.13 ^a^	547.31 ± 23.94 ^a^	973.62 ± 31.42 ^b^	<0.001
Ser	56.97 ± 4.13 ^a^	101.13 ± 3.75 ^c^	79.23 ± 7.74 ^b^	<0.001
Glu + Gln	727.64 ± 17.18 ^a^	771.24 ± 73.26 ^a^	998.65 ± 70.34 ^b^	0.003
Gly	N.d.	1.91 ± 0.06 ^b^	0.87 ± 0.08 ^a^	<0.001
Ala	2.57 ± 0.30 ^a^	2.99 ± 0.11 ^a^	5.70 ± 0.77 ^b^	<0.001
Pro	93.46 ± 4.41 ^a^	122.05 ± 3.99 ^b^	131.02 ± 5.20 ^b^	<0.001
Tyr	714.63 ± 11.71 ^a^	820.28 ± 28.47 ^b^	923.00 ± 47.14 ^c^	<0.001
**Total aa**	5200.74 ± 195.91 ^a^	5751.00 ± 204.15 ^b^	6948.93 ± 125.01 ^c^	<0.001
**Minerals** **(mg/100 g fw)**				
Ca	1.03 ± 0.02 ^b^	1.17 ± 0.07 ^c^	0.89 ± 0.01 ^a^	<0.001
Cu	0.31 ± 0.00 ^a^	0.32 ± 0.02 ^ab^	0.34 ± 0.01 ^b^	0.036
Fe	0.98 ± 0.01 ^a^	1.13 ± 0.05 ^a^	2.35 ± 0.12 ^b^	<0.001
K	3.37 ± 0.03 ^a^	3.72 ± 0.07 ^b^	3.81 ± 0.21 ^b^	0.013
Mg	1.03 ± 0.03 ^a^	1.11 ± 0.05 ^ab^	1.13 ± 0.00 ^b^	0.017
Mn	0.17 ± 0.02 ^a^	0.15 ± 0.01 ^a^	0.17 ± 0.00 ^a^	N.s.
Na	2.04 ± 0.09 ^a^	2.23 ± 0.04 ^b^	2.11 ± 0.00 ^ab^	0.010
Zn	0.44 ± 0.01 ^a^	0.53 ± 0.01 ^b^	0.56 ± 0.01 ^c^	<0.001
**Total minerals**	9.36 ± 0.10 ^a^	10.36 ± 0.30 ^b^	11.35 ± 0.33 ^c^	<0.001

Values are presented as the mean ± SD (*n* = 3) for the content of each nutritional composition at different locations. Mean values followed by different lowercase letters report significant differences between distinct locations, according to the analysis of variance (ANOVA) and Tukey’s multiple range test. N.s., not significant. N.d., not detected.

**Table 5:** Comparison between different locations throughout four years and between different years in the different locations. The values presented in each location were obtained by calculating the mean of values recorded in each location in the 4 years of study (2016–2019). The values for each year (Y2016, Y2017, Y2018, Y2019) were obtained by calculating the mean values of the locations in each year.

Nutritional Composition	Location				Year				
	Location 1	Location 2	Location 3	*p*-Value	Y2016	Y2017	Y2018	Y2019	*p*-Value
**Energy value** **(kcal/100 g)**	168.38 ± 22.78 ^a^	165.63 ± 16.92 ^a^	150.51 ± 27.3 ^a^	N.s.	151.16 ± 12.95 ^b^	191.63 ± 2.74 ^d^	168.48 ± 10.54 ^c^	134.77 ± 11.1 ^a^	<0.001
**Basic nutrients** **(g/100 g fw)**									
Ash	1.68 ± 0.25 ^a^	1.59 ± 0.25 ^a^	1.57 ± 0.32 ^a^	N.s.	1.50 ± 0.11 ^b^	2.03 ± 0.05 ^c^	1.58 ± 0.13 ^b^	1.34 ± 0.02 ^a^	<0.001
Moisture	61.83 ± 4.91 ^a^	61.89 ± 4.08 ^a^	64.72 ± 5.90 ^a^	N.s.	65.25 ± 3.55 ^c^	55.94 ± 0.22 ^a^	61.71 ± 1.29 ^b^	68.36 ± 1.44 ^d^	<0.001
Protein	4.30 ± 0.71 ^a^	4.12 ± 1.00 ^a^	4.31 ± 1.41 ^a^	N.s.	3.00 ± 0.24 ^a^	4.28 ± 0.46 ^b^	4.01 ± 0.13 ^b^	5.69 ± 0.64 ^c^	<0.001
Fat	4.61 ± 1.44 ^b^	3.87 ± 0.74 ^ab^	3.24 ± 0.50 ^a^	0.007	2.92 ± 0.47 ^a^	4.23 ± 0.89 ^b^	4.29 ± 1.32 ^b^	4.19 ± 1.07 ^b^	0.015
Carbohydrates	27.58 ± 4.82 ^a^	28.53 ± 5.00 ^a^	26.15 ± 5.59 ^a^	N.s.	27.34 ± 3.57 ^b^	33.52 ± 1.35 ^c^	28.41 ± 0.29 ^b^	20.42 ± 0.86 ^a^	<0.001
Dietary Fibre	9.28 ± 1.23 ^a^	8.59 ± 1.37 ^a^	9.90 ± 1.47 ^a^	N.s.	9.43 ± 0.94 ^ab^	8.97 ± 0.53 ^a^	10.50 ± 1.53 ^b^	8.14 ± 1.46 ^c^	0.002
Soluble Sugar	17.79 ± 2.62 ^a^	17.87 ± 2.29 ^a^	16.88 ± 3.41 ^a^	N.s.	16.65 ± 1.69 ^b^	21.34 ± 0.20 ^d^	17.88 ± 0.33 ^c^	14.18 ± 0.77 ^a^	<0.001
**Amino acids (mg/100 g fw)**									
**Essential aminoacids**									
His	95.08 ± 95.51 ^a^	106.02 ± 102.25 ^a^	108.57 ± 128.82 ^a^	N.s.	33.83 ± 3.15 ^a^	55.00 ± 13.72 ^a^	41.60 ± 4.09 ^a^	282.46 ± 35.10 ^b^	<0.001
Arg	438.98 ± 453.95 ^a^	461.40 ± 501.88 ^a^	499.23 ± 605.71 ^a^	N.s.	145.90 ± 22.99 ^a^	200.82 ± 25.94 ^a^	192.09 ± 39.08 ^a^	1327.30 ± 144.17 ^b^	<0.001
Thr	74.96 ± 19.36 ^a^	81.56 ± 24.72 ^a^	71.11 ± 29.04 ^a^	N.s.	48.06 ± 5.29 ^a^	81.43 ± 13.92 ^b^	70.72 ± 18.51 ^b^	103.29 ± 15.96 ^c^	<0.001
Val	95.97 ± 24.96 ^a^	122.70 ± 27.44 ^a^	111.42 ± 40.12 ^a^	N.s.	77.28 ± 9.46 ^a^	120.33 ± 8.97 ^b^	109.27 ± 16.98 ^ab^	133.24 ± 47.47 ^b^	<0.001
Lys	385.97 ± 529.12 ^a^	398.37 ± 514.16 ^a^	386.09 ± 566.35 ^a^	N.s.	89.24 ± 30.87 ^a^	113.50 ± 24.86 ^a^	82.80 ± 16.94 ^a^	1275.00 ± 112.33 ^b^	<0.001
Ileu	79.17 ± 22.18 ^a^	73.10 ± 9.96 ^a^	82.47 ± 24.60 ^a^	N.s.	60.87 ± 6.30 ^a^	91.53 ± 12.52 ^b^	82.77 ± 16.60 ^b^	77.82 ± 26.07 ^ab^	0.005
Leu	177.54 ± 39.23 ^a^	172.74 ± 25.86 ^a^	171.78 ± 33.31 ^a^	N.s.	147.00 ± 12.24 ^a^	212.07 ± 14.98 ^c^	181.48 ± 25.88 ^b^	155.53 ± 25.47 ^ab^	<0.001
Phe	116.51 ± 25.12 ^a^	112.74 ± 18.61 ^a^	110.24 ± 24.56 ^a^	N.s.	99.94 ± 8.76 ^a^	141.43 ± 9.91 ^c^	121.37 ± 13.48 ^b^	89.93 ± 8.24 ^a^	<0.001
Trp	15.83 ± 5.27 ^a^	15.51 ± 4.43 ^a^	15.72 ± 2.72 ^a^	N.s.	13.40 ± 0.83 ^b^	18.54 ± 1.77 ^c^	20.01 ± 1.22 ^c^	10.80 ± 2.67 ^a^	<0.001
**Non-essential aminoacids**									
Asp + Asn	673.08 ± 169.92 ^a^	701.07 ± 118.00 ^a^	678.74 ± 201.85 ^a^	N.s.	582.99 ± 106.78 ^a^	754.35 ± 92.32 ^a^	739.50 ± 128.79 ^a^	660.34 ± 240.56 ^a^	N.s.
Ser	81.48 ± 27.10 ^a^	83.44 ± 14.36 ^a^	67.56 ± 14.54 ^a^	N.s.	59.67 ± 10.22 ^a^	91.43 ± 12.45 ^b^	79.75 ± 24.02 ^ab^	79.11 ± 19.71 ^ab^	0.005
Glu + Gln	931.57 ± 220.21 ^a^	874.73 ± 97.66 ^a^	889.50 ± 150.12 ^a^	N.s.	746.78 ± 79.02 ^a^	1045.3 ± 121.88 ^c^	969.80 ± 113.99 ^bc^	832.5 ± 136.14 ^ab^	<0.001
Gly	82.29 ± 57.52 ^a^	100.13 ± 68.66 ^a^	65.40 ± 41.37 ^a^	N.s.	73.04 ± 8.22 ^b^	134.36 ± 29.73 ^c^	122.10 ± 31.01 ^c^	0.93 ± 0.83 ^a^	<0.001
Ala	89.32 ± 56.86 ^a^	87.20 ± 53.03 ^a^	73.92 ± 43.47 ^a^	N.s.	87.60 ± 9.63 ^b^	123.75 ± 13.20 ^c^	118.82 ± 21.28 ^c^	3.76 ± 1.53 ^a^	<0.001
Pro	148.10 ± 49.04 ^a^	164.65 ± 56.94 ^a^	152.75 ± 61.65 ^a^	N.s.	106.16 ± 22.15 ^a^	235.68 ± 23.63 ^c^	163.32 ± 4.10 ^b^	115.51 ± 17.44 ^a^	<0.001
Tyr	942.37 ± 186.46 ^a^	941.82 ± 135.65 ^a^	909.80 ± 117.88 ^a^	N.s.	791.11 ± 48.56 ^a^	1053.60 ± 69.27 ^b^	1061.30 ± 71.47 ^b^	819.3 ± 94.52 ^a^	<0.001
**Total aa**	4428.23 ± 889.97 ^a^	4497.15 ± 920.05 ^a^	4394.3 ± 1806.21 ^a^	N.s.	3162.87 ± 369.78 ^a^	4473.14 ± 232.65 ^b^	4156.67 ± 553.88 ^b^	5966.89 ± 893.87 ^c^	<0.001
**Minerals (mg/100 g fw)**									
Ca	0.82 ± 0.14 ^a^	0.85 ± 0.21 ^a^	0.80 ± 0.10 ^a^	N.s.	0.79 ± 0.07 ^b^	0.81 ± 0.04 ^b^	0.66 ± 0.04 ^a^	1.03 ± 0.13 ^c^	<0.001
Cu	0.43 ± 0.10 ^a^	0.38 ± 0.04 ^a^	0.39 ± 0.12 ^a^	N.s.	0.36 ± 0.07 ^ab^	0.41 ± 0.04 ^b^	0.51 ± 0.10 ^c^	0.32 ± 0.01 ^a^	<0.001
Fe	1.55 ± 0.46 ^a^	1.52 ± 0.30 ^a^	1.72 ± 0.40 ^a^	N.s.	1.34 ± 0.10 ^a^	1.77 ± 0.09 ^b^	1.78 ± 0.26 ^b^	1.48 ± 0.65 ^a^	0.030
K	4.98 ± 1.06 ^a^	4.80 ± 0.79 ^a^	4.32 ± 0.91 ^a^	N.s.	4.40 ± 0.69 ^b^	5.86 ± 0.14 ^c^	4.94 ± 0.57 ^b^	3.63 ± 0.23 ^a^	<0.001
Mg	1.01 ± 0.09 ^a^	0.98 ± 0.09 ^a^	0.96 ± 0.13 ^a^	N.s.	0.87 ± 0.06 ^a^	1.01 ± 0.03 ^bc^	0.96 ± 0.10 ^b^	1.09 ± 0.05 ^c^	<0.001
Mn	0.18 ± 0.02 ^b^	0.15 ± 0.03 ^a^	0.18 ± 0.02 ^ab^	0.024	0.18 ± 0.01 ^a^	0.17 ± 0.01 ^a^	0.17 ± 0.05 ^a^	0.16 ± 0.01 ^a^	N.s.
Na	2.47 ± 0.32 ^a^	2.61 ± 0.33 ^a^	2.59 ± 0.82 ^a^	N.s.	2.25 ± 0.18 ^ab^	3.24 ± 0.54 ^c^	2.61 ± 0.26 ^b^	2.13 ± 0.10 ^a^	<0.001
Zn	0.65 ± 0.19 ^a^	0.55 ± 0.06 ^a^	0.53 ± 0.14 ^a^	N.s.	0.46 ± 0.06 ^a^	0.74 ± 0.13 ^b^	0.59 ± 0.14 ^a^	0.51 ± 0.06 ^a^	<0.001
**Total minerals**	12.08 ± 2.21 ^a^	11.83 ± 1.38 ^a^	11.5 ± 2.08 ^a^	N.s.	10.64 ± 1.08 ^a^	14.00 ± 0.37 ^c^	12.21 ± 1.42 ^b^	10.36 ± 1.00 ^a^	<0.001

Values are presented as the mean ± SD for the content of each nutritional component at different locations and in different years. Mean values followed by different lowercase letters report significant differences between distinct locations or years, according to the Analysis of Variance (ANOVA) and Tukey’s multiple range test. N.s., not significant.

## Data Availability

All data are contained in this article and Supplementary Materials.

## Supplementary Materials

The following supporting information can be downloaded at: https://www.mdpi.com/article/10.3390/foods12050973/s1, Table S1. Meteorological data concerning the monthly accumulated precipitation (mm), maximum temperature (°C), minimum temperature (°C), and mean temperature (°C) in the years 2016, 2017, 2018, and 2019. Data were obtained from the E-OBS observational gridded dataset [26]. Daily air temperatures and precipitation were extracted from the gridbox corresponding to Vila Real. The maximum and minimum temperatures displayed in each month correspond to the mean value of the maximum and minimum temperatures recorded on each day, respectively. The mean temperature corresponds to the mean value between the maximum and minimum temperature; Figure S1. Mean monthly temperature (°C) and rainfall (mm) in 2016, 2017, 2018, and 2019 at Vila Real region of Northern Portugal.
