# Supplementary material for: Prunus lusitanica L. Fruits: A Promising Underexploited Source of Nutrients with Potential Economic Value
Source: Foods. 2023 Feb 24;12(5):973. doi: 10.3390/foods12050973 (PMC10001125; doi:10.3390/foods12050973)
Supplement: Supplementary file 1 [file foods-12-00973-s001.zip › foods-2195532-supplementary.pdf]

Supplementary material

Table S1. Meteorological data concerning the monthly accumulated precipitation (mm), maximum temperature (°C), minimum temperature (°C), and mean temperature (°C) in the years 2016, 2017, 2018, and 2019. Data were obtained from the E-OBS observational gridded dataset [26]. Daily air temperatures and precipitation were extracted from the gridbox corresponding to Vila Real. The maximum and minimum temperatures displayed in each month correspond to the mean value of the maximum and minimum temperatures recorded on each day respectively. The mean temperature corresponds to the mean value between the maximum and minimum temperature.

	Accumulated Precipitation (mm)				Maximum temperature (°C)			
**Month**	**2016**	**2017**	**2018**	**2019**	**2016**	**2017**	**2018**	**2019**
**Jan**	312.60	40.90	84.50	47.00	10.44	9.73	10.16	10.30
**Feb**	203.50	142.90	84.50	30.30	10.28	12.58	9.74	13.95
**Mar**	101.00	52.70	328.60	46.10	12.42	15.09	10.13	16.39
**Apr**	209.80	9.70	113.60	144.90	14.93	21.29	16.14	15.37
**May**	125.60	70.50	63.20	9.10	18.33	22.81	20.44	22.51
**Jun**	19.20	5.70	107.50	26.90	25.20	28.52	23.73	21.72
**Jul**	0.00	15.80	8.10	8.00	31.58	29.61	26.79	28.57
**Aug**	13.00	2.30	0.00	21.80	31.52	30.06	31.77	27.41
**Sep**	27.80	0.00	18.60	18.70	27.38	25.45	29.78	24.74
**Oct**	89.60	25.00	45.80	92.50	20.01	24.75	19.62	18.28
**Nov**	124.40	58.00	205.80	184.40	12.56	15.03	11.97	10.62
**Dec**	47.90	139.00	64.20	227.50	9.78	10.20	11.35	10.04

	Minimum temperature (°C)				Mean temperature (°C)			
**Month**	**2016**	**2017**	**2018**	**2019**	**2016**	**2017**	**2018**	**2019**
**Jan**	4.89	0.70	3.00	1.25	7.66	5.22	6.58	5.78
**Feb**	2.95	4.54	0.83	3.38	6.61	8.56	5.28	8.66
**Mar**	2.85	5.20	2.99	4.60	7.64	10.15	6.56	10.49
**Apr**	5.88	7.30	6.66	6.03	10.40	14.29	11.40	10.70
**May**	8.64	10.69	8.97	8.72	13.48	16.75	14.70	15.61
**Jun**	11.91	14.28	12.62	8.94	18.55	21.40	18.18	15.33
**Jul**	15.65	14.12	14.15	13.51	23.62	21.86	20.47	21.04
**Aug**	15.33	14.14	15.45	13.26	23.42	22.10	23.61	20.34
**Sep**	12.78	10.77	14.80	11.62	20.08	18.11	22.29	18.18
**Oct**	9.80	10.55	8.77	8.72	14.90	17.65	14.20	13.50
**Nov**	4.84	4.15	6.05	5.14	8.70	9.59	9.01	7.88
**Dec**	3.04	2.39	4.87	3.41	6.41	6.30	8.11	6.72

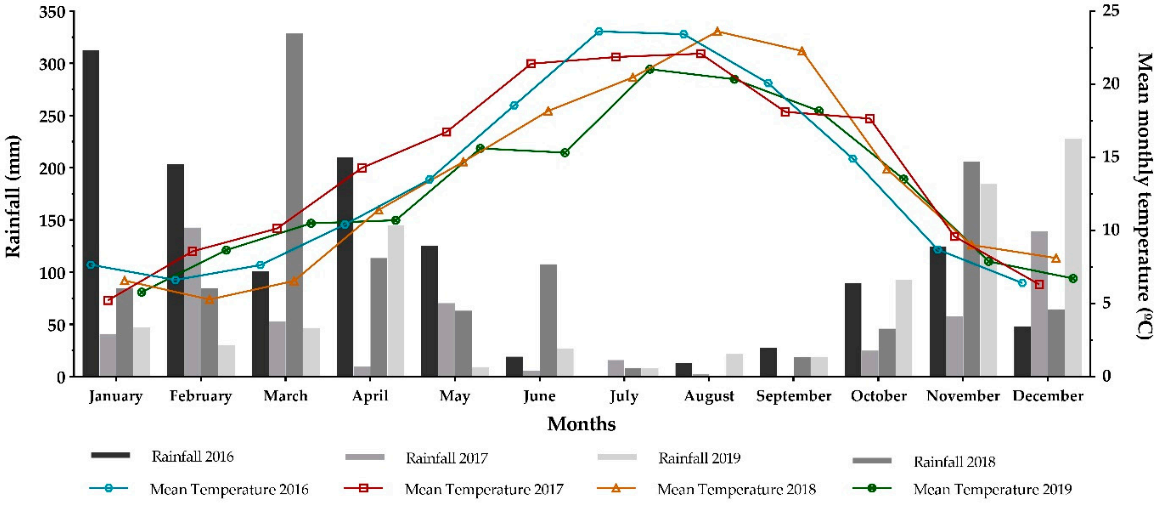

Figure S1. Average monthly temperature (°C) and accumulated monthly rainfall (mm) in 2016, 2017, 2018, and 2019 at Vila Real region, Northern Portugal.
